# Integrating Land-Cover Dynamics and Management Records for Recovery Monitoring of Hainan Eld’s Deer in Datian National Nature Reserve

**DOI:** 10.3390/ani16193086

**Published:** 2026-09-30

**Authors:** Tianao Chen, Chenxingyu Duan, Chuan Chen, Xiaoxian Liu, Yiting Zheng, Xiaofeng Luan

**Affiliations:** School of Ecology and Nature Conservation, Beijing Forestry University, Beijing 100083, China; chentianao@bjfu.edu.cn (T.C.); chenxingyuduan@bjfu.edu.cn (C.D.); riverschen1998@163.com (C.C.); liuxiaoxian0305@bjfu.edu.cn (X.L.); yitingzheng@bjfu.edu.cn (Y.Z.)

**Keywords:** Hainan Eld’s deer, Datian Nature Reserve, land-cover change, recovery monitoring, protected-area management, management fence, management records

## Abstract

Datian National Nature Reserve is a principal conservation site for the endangered Hainan Eld’s deer (*Rucervus eldii hainanus*). We integrated annual 10 m land-cover maps (2017–2025) as well as the statutory reserve redline, management fence, external ring and reserve management records to examine how these complementary sources can support recovery monitoring at the protected-area scale. Inside the redline, the combined area of trees, grass and shrub/scrub declined during 2017–2021 and partially recovered during 2021–2025. An exploratory land-cover screening index changed from 0.675 to 0.666 and then to 0.667, showing modest variation under the specified rule-based scoring scheme. External rings contained higher proportions of crops and built area and were treated as surrounding land-use context rather than a measured edge-effect gradient. Available annual monitoring counts fluctuated between 336 and 425 individuals, and two releases added 93 deer after the respective annual surveys. Probable python predation accounted for 68.3% of recorded deaths during 2020–2025; attributions were based on available carcass evidence and field observations. Systematic occurrence records and spatially complete locations for releases and mortality events were unavailable, so fine-scale habitat selection, release-site suitability and mortality hotspots could not be assessed. This assessment provides a practical baseline for recovery monitoring, whereas assessing habitat use and demographic processes requires dedicated field data.

## Simple Summary

Hainan Eld’s deer is an endangered deer restricted to Hainan Island, China. Datian National Nature Reserve is its principal protected site, but incomplete animal-location records make it difficult to assess habitat use and the causes of population change directly. We combined annual land-cover maps from 2017 to 2025 with reserve boundaries, management records, releases and recorded causes of death. Farmland and built land were much more common around the reserve than within it. Counts varied among survey years, and two releases occurred after the surveys in 2019 and 2024. Probable python predation was the most frequently recorded cause among detected deaths. The combined mapped vegetation area changed little overall, but grass cover declined. The combined records provide a practical baseline for recovery monitoring while showing why mapped land cover, animal counts and management events need to be interpreted separately. More standardized field records are still needed to assess habitat use and the processes underlying population change.

## 1. Introduction

Large ungulates are important components of terrestrial ecosystems and are often used as flagship or surrogate taxa because their persistence is closely associated with habitat integrity, landscape connectivity and reduced human disturbance [1,2]. Endangered ungulates often require relatively large areas, have limited demographic recovery potential and are sensitive to human disturbance [2]. Therefore, protected areas (PAs) have been widely recognized as one of the most important tools for their conservation and recovery [3,4]. However, protected status alone does not ensure effective conservation and may depend on boundary control, surrounding land use, fragmentation and sustained management investment [4,5].

Assessing the recovery of endangered ungulates inside PAs remains a challenge. Reintroduction studies emphasize the need to monitor survival, reproduction and persistence after release [6,7,8,9]. Survey design and imperfect detection also affect the interpretation of abundance and occupancy estimates [10,11]. We therefore assessed reserve-scale monitoring status through land-cover context, an exploratory land-cover screening index, management input and recorded causes of death. This is particularly important for populations living in small, clearly bounded PAs under long-term management, because monitoring counts may reflect demographic change, survey design, supplementation, mortality and the amount of usable space available to the animals.

Land-cover change provides repeatable spatial evidence for PA-scale assessment [12,13]. Previous PA-scale assessments have often relied on field surveys, vegetation plots, transect or camera-trap records, vegetation indices such as the normalized difference vegetation index (NDVI), and medium-resolution land-cover or forest-cover change products to describe habitat condition and human disturbance [13,14]. These approaches are useful for linking habitat change with conservation outcomes, but they may be limited when small PAs require repeated, fine-scale and comparable annual monitoring. In addition, the availability of near-real-time 10 m land-cover products (LCPs), from which annual composites can be generated, has made it possible to monitor small PAs at a finer spatial scale, where long-term occurrence data are incomplete [15]. LCPs provide a consistent framework for evaluating mapped land-cover conditions and external land-use context, but they do not measure habitat quality or realized habitat use, and field habitat surveys cannot be replaced [13,15,16].

However, the area inside the formal boundary is only one part of the picture. Here, the reserve redline is retained as a local management term and refers to the statutory or planning boundary of Datian Nature Reserve, rather than China’s national Ecological Conservation Redline system. In this study, land-cover change inside the redline describes the mapped conservation unit, while the management fence indicates the area enclosed by the supplied fence boundary. External rings describe the surrounding agricultural and built-up matrix [17,18]. Human pressure can remain substantial even within protected areas [19]. Assessments should therefore consider both the reserve and its surrounding land-use context [17,18]. Comparing the legal boundary, practical management fence and external rings within one analytical framework therefore provides a clearer view of internal land cover and surrounding land use. This comparison provides a spatial context for interpreting reserve-level management records; individual observations could not be spatially matched to land-cover classes.

In recent years, China has strengthened biodiversity conservation by mainstreaming biodiversity into national planning and improving its conservation and protected-area management systems [20]. Against this broader conservation background, Hainan Eld’s deer (HED), an endangered flagship deer taxon on Hainan Island, has undergone substantial historical recovery under protection [21,22]. Datian National Nature Reserve (DNNR) has played a central role in this recovery. Previous studies have examined the population history of HED, genetic bottlenecks, habitat management, post-relocation habitat use and broader conservation priorities [21,22,23,24]. These studies have shown that HED recovered from an extremely small remnant population under reserve protection, but genetic bottlenecks, habitat limitation, human disturbance and the need for suitable lowland habitats remain important conservation concerns [21,22,23]. The present assessment brings annual land-cover dynamics, formal reserve boundaries, practical management fences, external land use and management records together at the reserve scale.

Against this background, we used a multi-source assessment to examine how land-cover dynamics and management records can jointly inform recovery monitoring in a small, intensively managed reserve [6,7,8,9]. Specifically, we asked (1) how land-cover composition and temporal change differed among the reserve redline, management fence and surrounding rings, and which transitions predominated inside the redline; (2) whether the reserve retained a greater proportion of vegetation-related cover and less built land than its surroundings; (3) whether the direction of temporal change remained consistent under the main and conservative score sets; and (4) how monitoring counts should be interpreted in relation to survey conditions, releases and recorded deaths. These questions concern the evidence available for recovery monitoring rather than direct tests of habitat selection or demographic mechanisms.

## 2. Materials and Methods

### 2.1. Study Area and Spatial Units

**Figure 1:**
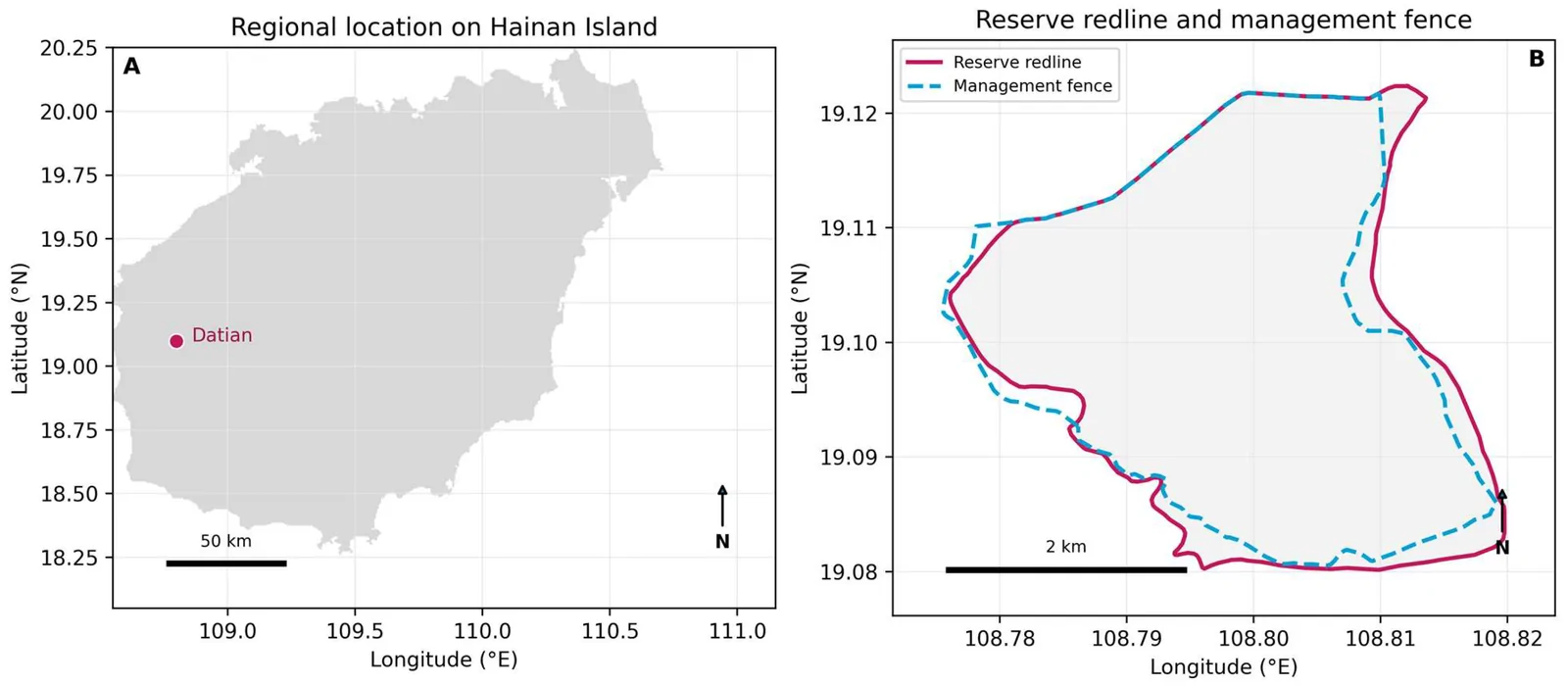
(**A**) Regional location of Datian National Nature Reserve on Hainan Island; (**B**) spatial relationship between the statutory reserve redline and management fence. Geographic coordinates, north arrows and scale bars are shown.

DNNR is located in western Hainan Island, China, and represents the core in situ conservation area for Hainan Eld’s deer (Figure 1). The study area comprised three spatial components: the statutory reserve redline, the management fence and external rings. The redline was used as the core spatial unit for legal protection, while the fence was added to represent the supplied practical management boundary. The area of the redline vector was approximately 1310 ha, and the effective area after overlaying it with the annual land-cover rasters was approximately 1309.87 ha. The effective raster-overlap area inside the fence was 1215.32 ha. External 0–1 km, 1–3 km and 3–5 km rings were created outside the redline to distinguish immediate, intermediate and broader surrounding land-use contexts.

**Figure 2:**
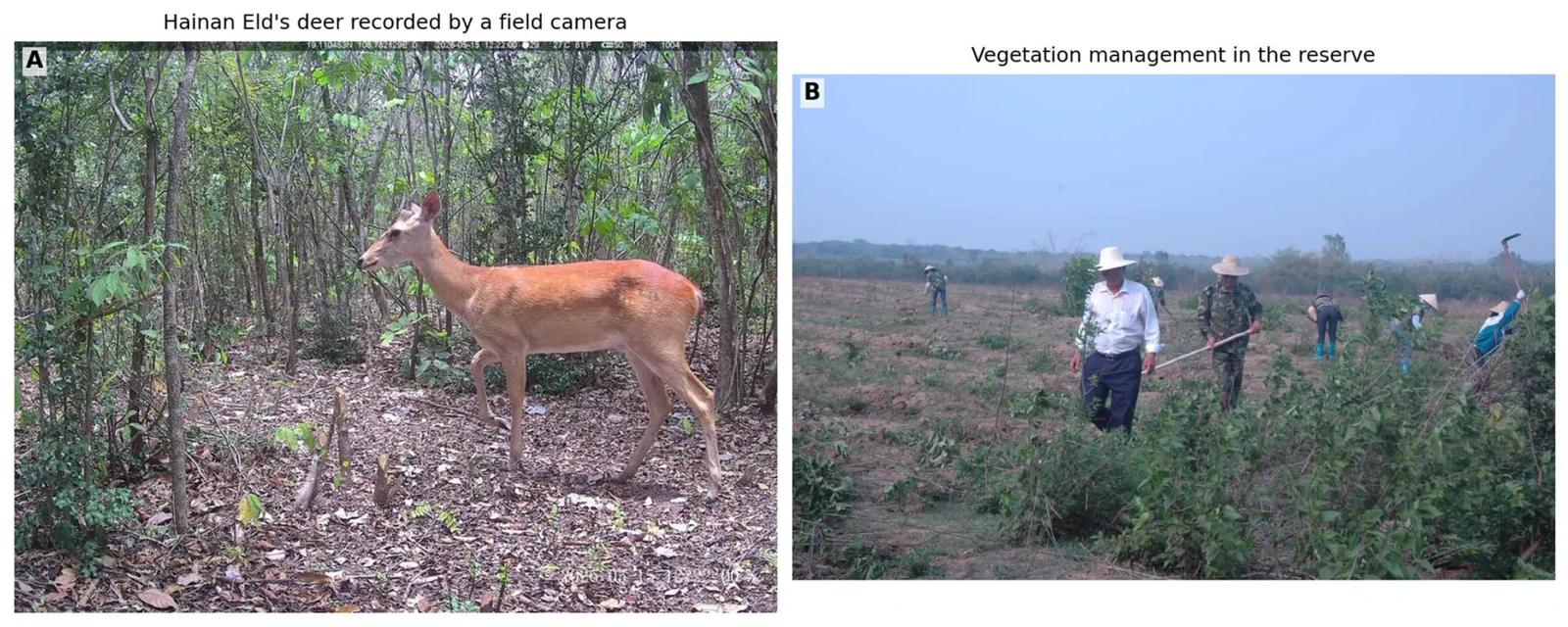
Representative reserve-supplied photographs. (**A**) Hainan Eld’s deer recorded by a field camera; (**B**) vegetation-management work. The photographs provide field context and were not used to estimate deer occurrence or calibrate the land-cover analysis.

The redline and the fence are spatially similar but conceptually different. The redline represents the legal or planning boundary, while the fence represents the boundary contained in the supplied management file. Including both layers helps avoid treating the administrative boundary and the practical management boundary as identical. Wong et al. [22] reported that Datian’s first 1 km^2^ enclosure was built in 1986 using a 2.2 m high wire fence and that the 13.14 km^2^ reserve was fully enclosed by 1995. Pan et al. [21] described a 2.8 m perimeter fence. These historical accounts do not establish a single design for all fence sections during 2017–2025. The available map was not accompanied by a segment-level record of dimensions, mesh, gates, damage or maintenance. Historical enclosure was therefore distinguished from contemporary integrity and biological permeability [25,26]. The three non-overlapping external distance bands were used as observation zones, rather than as evidence of deer movement ranges, disturbance thresholds or a measured edge effect. The same supplied redline and fence geometries were used for all years as fixed analytical boundaries; this does not establish that fence location or condition remained unchanged throughout 2017–2025. Representative reserve-supplied photographs of a deer and vegetation-management work are provided as field context (Figure 2).

### 2.2. Data Sources and Preprocessing

Dynamic World V1 was used as the source of annual land-cover data. It is a near-real-time 10 m global land-use/land-cover product derived from Sentinel-2 imagery [15]. Processing was conducted in Google Earth Engine [27]. For each year, we applied a mode reducer to the label band of GOOGLE/DYNAMICWORLD/V1 to obtain the most frequent class among available observations. The script used 1 January as the start date and 31 December as the exclusive end date; observations from 31 December were therefore not included. The product’s existing cloud and shadow masks were retained, and no additional class-probability threshold was applied. Class areas were calculated with ee.Image.pixelArea() at a nominal 10 m analysis scale, and label maps were exported in EPSG:4326. The nine classes were water, trees, grass, flooded vegetation, crops, shrub and scrub, built area, bare ground, and snow and ice. These classes were treated as remotely sensed land-cover labels rather than field-verified fine habitat types. The data sources, temporal coverage and analytical uses are summarized in Table 1.

The annual mode composite was chosen as a transparent and reproducible summary that reduced reliance on any single acquisition. In tropical Hainan, crop phenology, cloud contamination and short-term vegetation changes can affect image-level classification. The resulting map represents the most frequent label among available observations; it can conceal seasonal variation and does not remove classification uncertainty. It should not be read as a field vegetation map.

### 2.3. Land-Cover Change, Transition Matrices and Exploratory Screening Index

For the redline, the fence and each external ring, annual area and proportion were calculated for each land-cover class. Changes were summarized for the periods 2017–2021, 2021–2025, and 2017–2025. Transition matrices were calculated for the area inside the reserve redline to identify dominant conversion pathways. Trees, grass, shrub and scrub, and crops were retained as separate mapped classes. Vegetation-related cover was defined as trees, grass, and shrub and scrub.

The exploratory land-cover screening index was calculated as the area-weighted mean score shown below, where Ai is the mapped area of class i and si is its assigned score. A conservative scoring scheme and an unweighted vegetation-related proportion were also applied. The index was used for reserve-scale sensitivity screening and was not interpreted as habitat suitability, occupancy, carrying capacity or probability of deer use. No ecological cutoff defines a high, low or acceptable value.

$$I=\frac{\sum_{i}\;A_{i}s_{i}}{\sum_{i}\;A_{i}}$$

The scores were designed for relative screening at the reserve scale rather than for estimating absolute habitat quality. Their ordering was informed by reported use of open foraging sites, shrub patches and forest-edge conditions [21,24], but the numerical values were chosen by the authors, not estimated from field observations. Hainan Eld’s deer can use a mosaic of open and wooded habitats. Crops received a lower score to represent human-managed cover, although published evidence shows that deer can use some agricultural habitats [21]. Dynamic World does not resolve the differences in crop type, forage or disturbance that influence such use. The conservative scheme reduced the main scores by 0.10 for water, trees, grass, flooded vegetation and shrub/scrub, and by 0.05 for crops and bare ground; built area and snow/ice were unchanged (Table 2). This tests one alternative score set, not the full uncertainty in class rankings. Direct class-area results were therefore given greater interpretive weight than the index.

### 2.4. Landscape Metrics

Landscape metrics were calculated for vegetation-related cover and included patch number, total area, mean patch area, largest patch area, largest patch index and edge density. Contiguous patches were identified using eight-neighbor connectivity, and all mapped patches were retained. Largest patch index was calculated relative to the total redline raster area. Edge density was calculated as the total length of edges separating vegetation-related cover from other classes or the reserve-mask boundary, divided by the total redline area in hectares. Cell areas and edge lengths were adjusted for latitude on the geographic-coordinate raster grid. All metrics were calculated in Python 3.12.14. They describe the spatial pattern of selected raster labels and are not estimates of functional connectivity [28].

### 2.5. Management Records and Interpretation

Management records were interpreted conservatively. Annual counts can be affected by survey days, survey method, weather, visibility and animal movement and thus were treated as monitoring counts rather than exact abundance estimates. Release and mortality records also lack complete spatial coordinates. They were therefore used to identify major management events and recorded causes of death, rather than to build a formal population-dynamic model. No annual birth, death or population growth rates were estimated.

Annual survey summaries were supplied through the Hainan Datian National Nature Reserve Administration. The reserve is the reported survey setting, but the summaries do not identify individual staff roles, exact transect routes or the area actually searched. Counts were available for 2017, 2018, 2019, 2021, 2024 and 2025. The recorded survey periods fell between March and May, with 11, 33, 36, 23, 37 and 19 survey days, respectively. The reported approaches were synchronous counts and line-transect work; infrared cameras and drone thermal imaging were also listed in 2024 and 2025. The summaries reported no repeat survey within a survey year; procedures for identifying repeat sightings within each survey were not documented. Observation-level detections, camera effort, weather, visibility and duplicate-count procedures were unavailable, so the annual totals could not be standardized for effort or detection probability [10,11].

Release records documented 46 deer in 2019 and 47 in 2024. Both releases occurred after the annual surveys, so released deer were not included in the corresponding same-year monitoring count. Spatially complete release locations and standardized post-release tracking were unavailable.

In the supplied records, abnormal events consisted mainly of detected deaths. For the cause summary, we used the entries tabulated under “number of deaths” in the reserve’s mortality or abnormal-event records. The denominator was the pooled total of those recorded deaths during 2020–2025. Reserve personnel classified deaths as probable python predation based on available carcass evidence and field observations; an independent diagnostic assessment was not available. Carcasses can go undetected, comparable search-effort records were unavailable, and coordinates were absent for most events. The proportion therefore describes recorded deaths, not a cause-specific mortality rate or the proportion of all deaths caused by pythons. The Hainan Datian National Nature Reserve Administration advised caution regarding disclosure of exact mortality counts, which have not been publicly released. We therefore report only the pooled cause proportion.

## 3. Results

### 3.1. Land-Cover Composition Inside the Redline

**Figure 3:**
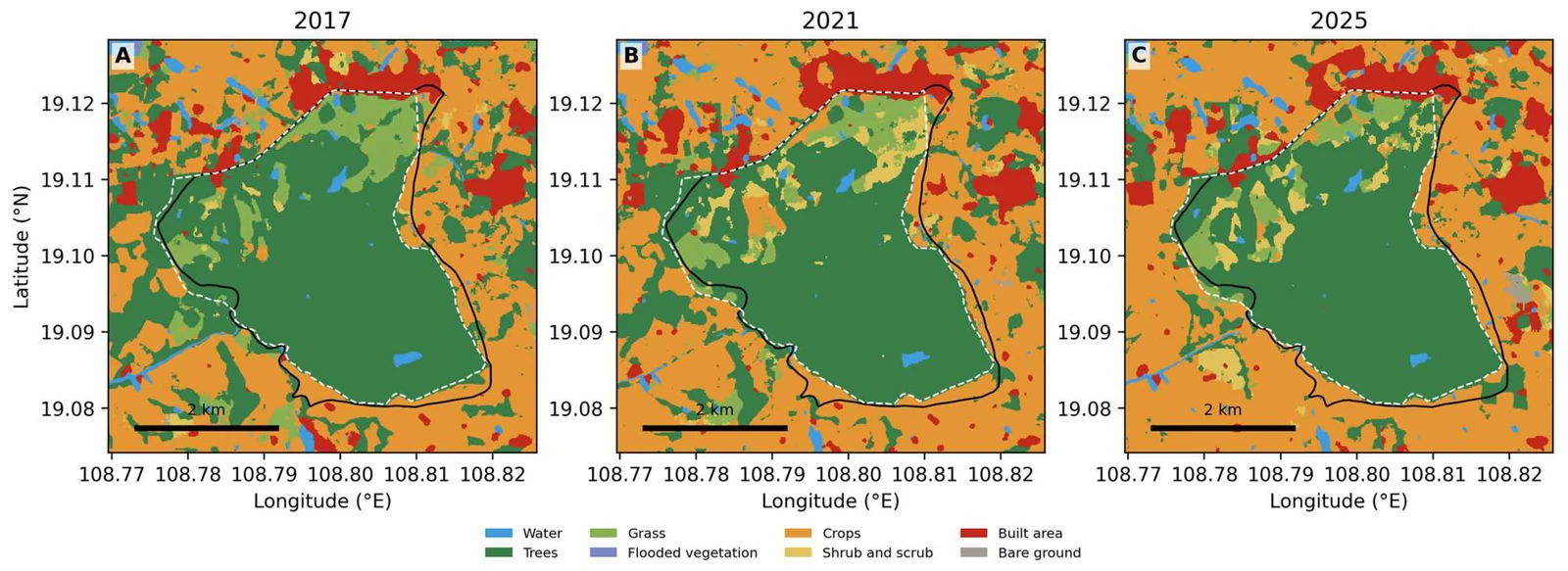
Spatial patterns of Dynamic World land-cover labels in Datian Nature Reserve in (**A**) 2017, (**B**) 2021 and (**C**) 2025, with the reserve redline and management fence overlaid. Each panel uses the same class colors and corresponds to the area statistics in Table 3. The solid black line denotes the reserve redline, and the dashed white line denotes the management fence.

**Figure 4:**
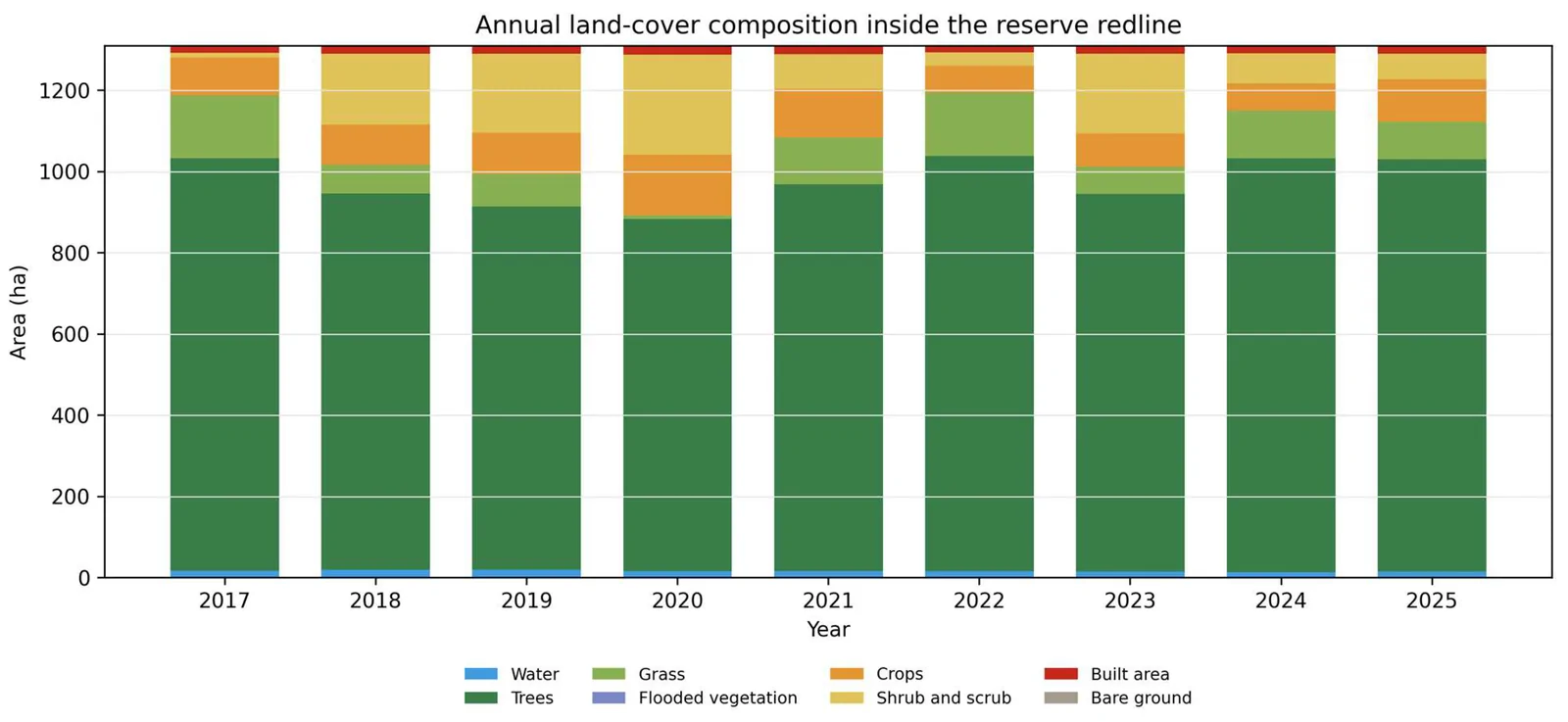
Changes in major land-cover composition inside the reserve redline during 2017–2025.

Land cover within the redline consisted mainly of trees, grass, crops, and shrub and scrub (Table 3; Figure 3 and Figure 4). In 2017, trees covered 1014.33 ha (77.4%), grass 155.70 ha (11.9%), and crops 93.19 ha (7.1%). By 2021, trees declined to 952.61 ha, crops increased to 120.59 ha, and shrub and scrub increased to 85.41 ha. In 2025, mapped tree cover increased to 1014.47 ha, crops declined to 104.60 ha, and grass and shrub and scrub covered 92.86 and 62.97 ha, respectively. Thus, the increase in vegetation-related cover by 2025 was driven by mapped tree cover rather than grass recovery.

### 3.2. Comparison Among the Redline, Fence and External Rings

**Figure 5:**
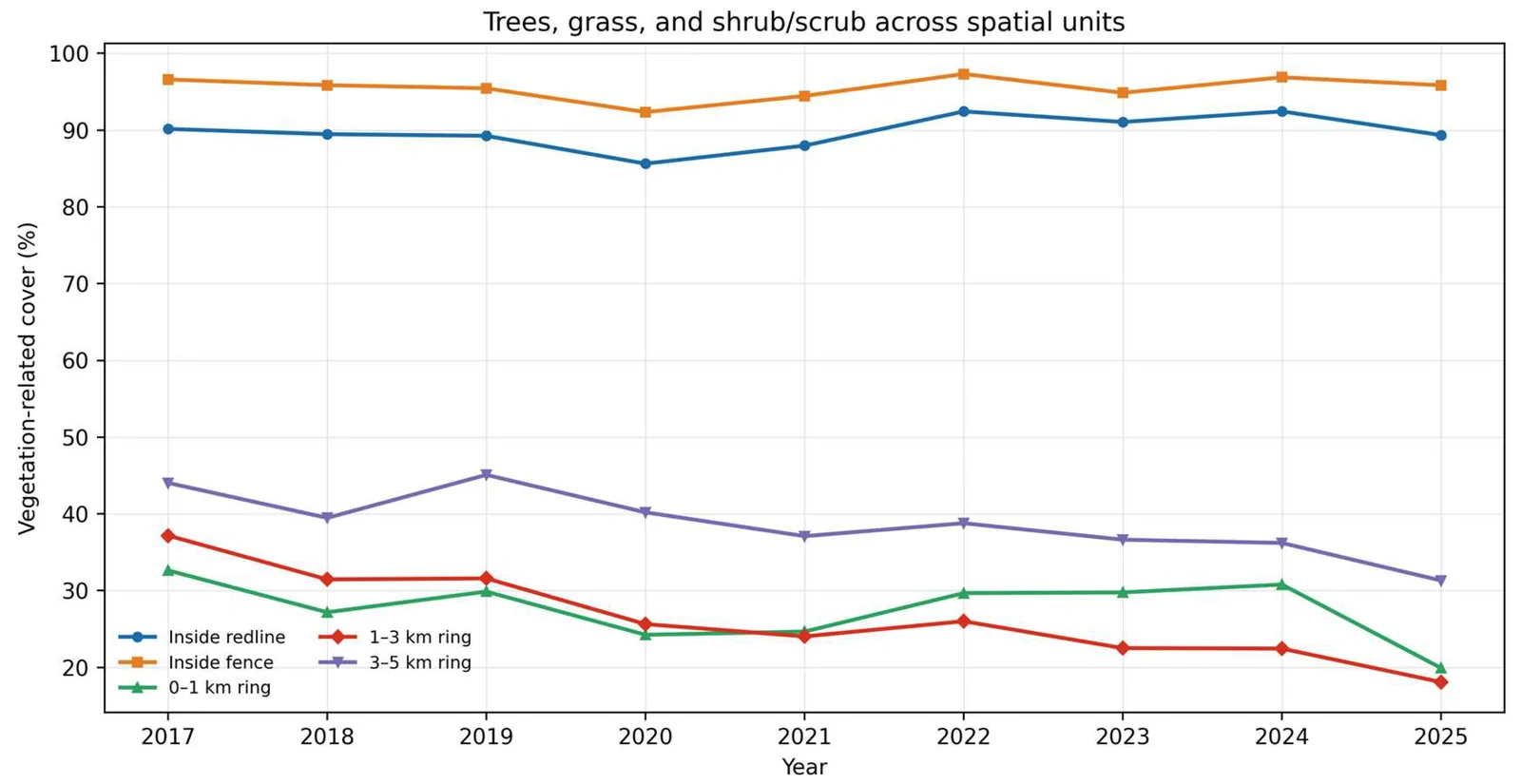
Changes in vegetation-related cover across spatial units during 2017–2025. External rings are presented as surrounding land-use context.

Land-cover composition in the external rings differed markedly from that within the redline (Table 4; Figure 5). In 2025, crops covered 1238.92 ha (66.08%) in the 0–1 km ring, 3873.36 ha (69.89%) in the 1–3 km ring, and 4322.01 ha (54.07%) in the 3–5 km ring. Built-up areas also occupied substantial proportions of the 0–1 km and 1–3 km rings, covering 215.37 and 462.09 ha, respectively. In contrast, the redline and fence retained higher proportions of trees. These statistics describe a strong internal–external land-use contrast; they do not measure an edge-effect response.

### 3.3. Transitions Involved Trees, Grass, Shrub and Crops

**Figure 6:**
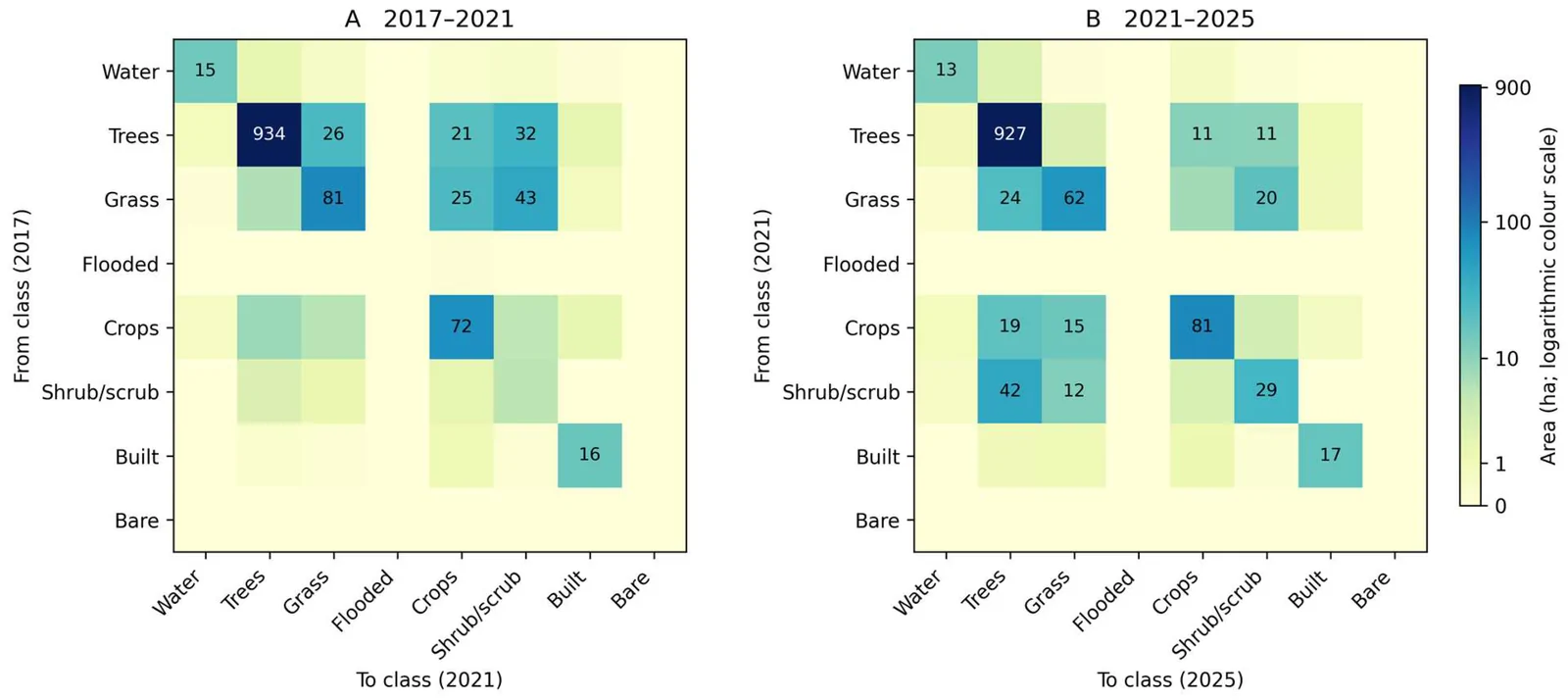
Land-cover transitions inside the redline during (**A**) 2017–2021 and (**B**) 2021–2025. Cell values are areas (ha); only values ≥10 ha are labeled, rounded to the nearest hectare. Colors use log10 (area in ha + 1) with a shared scale. Unlabeled cells are not necessarily zero.

Transition matrices showed that land-cover change involved multiple vegetation classes (Table 5; Figure 6). During 2017–2021, 21.01 ha of tree cover was converted to crops, 42.95 ha of grass to shrub and scrub, and 31.59 ha of trees to shrub and scrub. During 2021–2025, 18.97 ha of crops and 41.74 ha of shrub and scrub were converted to tree cover. The matrices report mapped class transitions only; they do not identify the ecological cause of change.

### 3.4. The Exploratory Screening Index Changed Modestly

**Figure 7:**
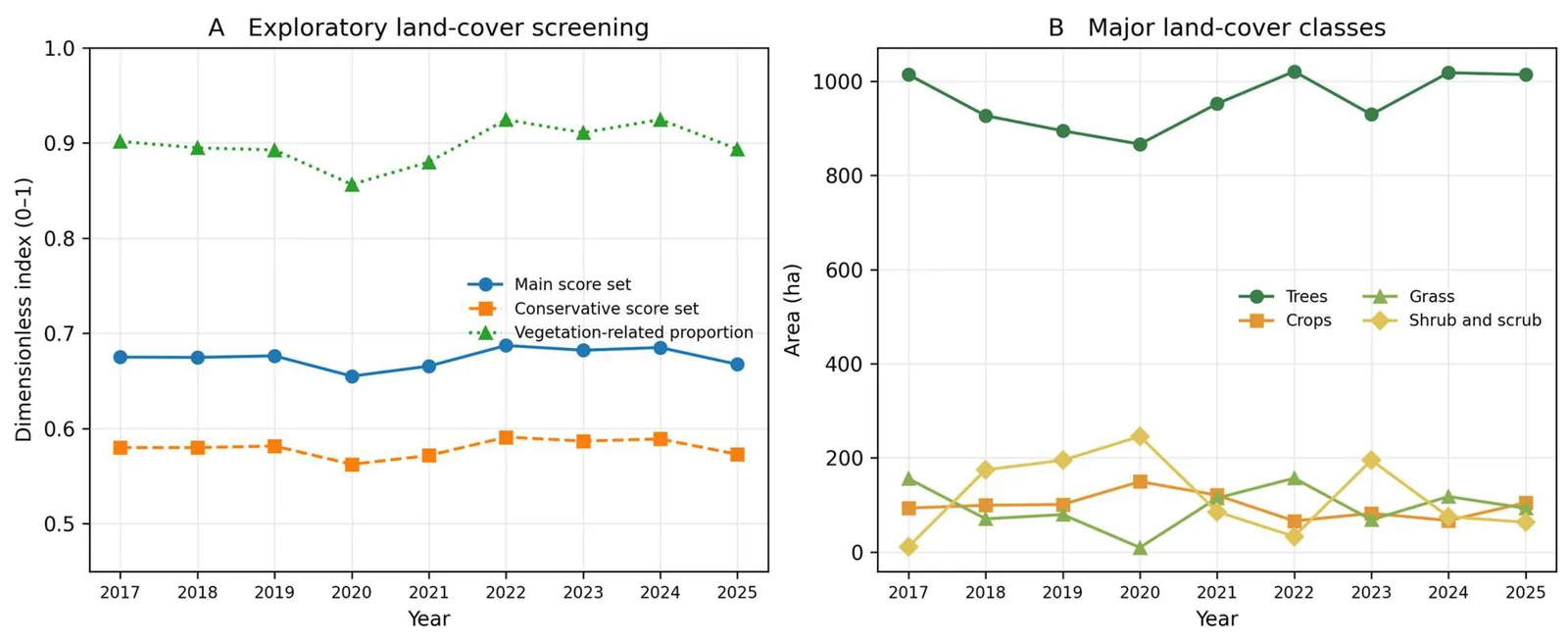
Annual changes in the exploratory land-cover screening index and dominant land-cover classes inside the reserve redline.

Under the main scoring scheme, the exploratory screening index inside the redline declined from 0.675 in 2017 to 0.666 in 2021 and was 0.667 in 2025 (Table 6; Figure 7). The conservative scheme showed the same temporal direction, changing from 0.580 to 0.572 and 0.573. The vegetation-related proportion declined from 0.902 to 0.880 and recovered to 0.893. These values show modest change under the declared scoring schemes; they are not estimates of habitat suitability.

### 3.5. Landscape Structure of Vegetation-Related Cover Fluctuated

When trees, grass, and shrub and scrub were grouped as vegetation-related cover, the total area decreased from 1180.92 ha in 2017 to 1152.45 ha in 2021 and recovered to 1170.29 ha in 2025 (Table 7). Patch number was 35, 33 and 20, while the largest-patch index was 90.02%, 87.82% and 89.15%, respectively. These raster metrics provide a coarse description of mapped spatial patterns.

### 3.6. Monitoring Counts, Post-Survey Releases and Recorded Causes of Death

**Figure 8:**
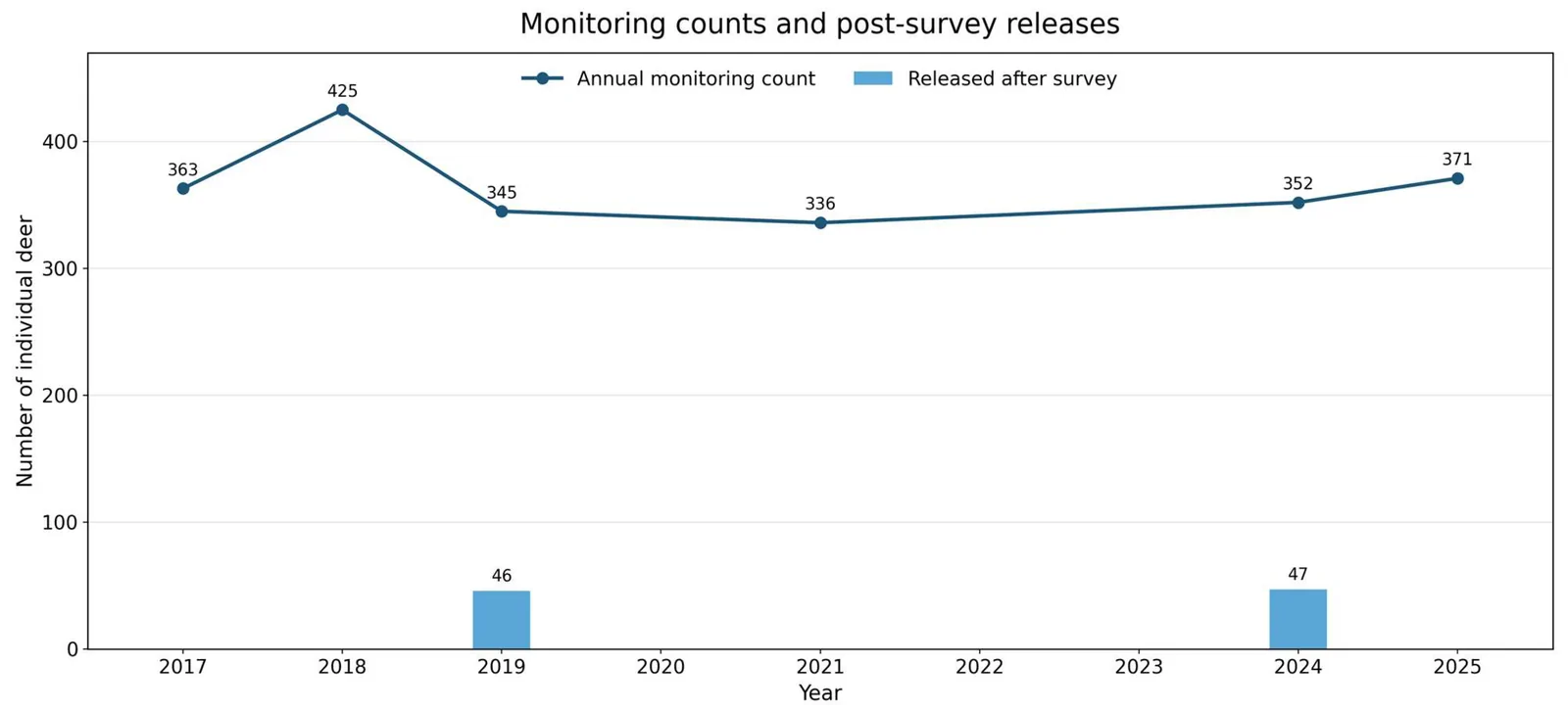
Monitoring counts and post-survey releases of Hainan Eld’s deer during 2017–2025. Missing count years indicate unavailable comparable values, not zero animals. Lines connect available observations only and do not represent estimates for unsurveyed years.

The monitored number of Hainan Eld’s deer fluctuated between 336 and 425 individuals during 2017–2025 (Table 8; Figure 8). The count was 425 in 2018, declined to 345 in 2019 and 336 in 2021, and was 352 and 371 in 2024 and 2025, respectively. Two post-survey release events added 93 individuals: 46 in 2019 and 47 in 2024. During 2020–2025, probable python predation accounted for 68.3% of recorded deaths and was the most frequently recorded probable cause. Exact mortality counts are not reported for the reasons given in Section 2.5.

## 4. Discussion

### 4.1. Interpretation of Land-Cover Dynamics and External Context

The redline was a mosaic of trees, grass, shrub and scrub, and crops. This is consistent with the range of vegetation types used by Hainan Eld’s deer in previous studies, although mapped classes alone cannot establish that these areas are used by deer [21,24]. Tree recovery alone should not be interpreted as habitat improvement without field verification of deer use and vegetation quality. Little net change in the combined area masked changes in vegetation composition: mapped grass cover declined from 155.70 ha in 2017 to 92.86 ha in 2025.

These staged changes may reflect a combination of real vegetation dynamics, short-term management or land-use disturbance, crop phenology, and classification uncertainty in open vegetation types; therefore, without paired climate and field vegetation data, they should not be attributed to climate change alone [13,15].

The contrast between the redline and external rings is consistent with protected-area studies showing that surrounding land use can affect ecological processes within reserves [17,18,29]. Across 60 tropical protected areas, Laurance et al. [29] found that environmental changes outside reserves were closely associated with ecological changes within them. Our ring analysis describes the surrounding land-cover context, but the available data do not test whether comparable biological responses occurred at Datian. Around Datian, crops occupied 66.08% of the 0–1 km ring in 2025, while built area covered 215.37 ha. In this setting, human activity near fields and buildings could alter deer activity or use of boundary habitat where animals encounter it. Agriculture may also provide forage, so its effects need not be uniformly negative. Relocated Hainan Eld’s deer at Chihao used some disturbed habitats but avoided intensively farmed areas [21]. This comparison identifies a plausible trade-off between forage and disturbance; it does not demonstrate the same response at Datian. Movement of water and associated materials across a reserve boundary is another possible pathway [17], but no local exposure measurements were available. The ring statistics therefore locate a contrasting land-use setting, not the penetration distance or magnitude of an edge effect.

Fencing may modify these relationships selectively. Conservation-fence studies show that a barrier can retain a target species while excluding some animals and remaining permeable to others [25,26]. At Datian, the initial enclosure was intended to reduce poaching and disturbance from dogs and livestock, while enclosure restricted natural deer dispersal [22]. This history supports a protective purpose, not a measured exclusion rate during our study. Smaller animals, climbing animals and flying species may cross barriers that impede deer; these are possibilities, not observations from our fence layer. Python predation records show that deer and pythons interacted but cannot distinguish predators already inside the enclosure from animals crossing the fence. Nor would an ordinary perimeter fence necessarily prevent disturbance from nearby human activity or cross-boundary water flows. The mapped fence therefore defines a management boundary, while its biological selectivity remains unmeasured. The difference between fence and redline areas is not evidence of gaps in the fence.

The similar temporal direction under the two scoring schemes provides a limited sensitivity check. It does not validate the scores or establish that other plausible rankings would yield the same result. Remote-sensing products can support repeated assessment but cannot replace field surveys or occurrence-based habitat models [13,15,16]. The result therefore concerns a rule-based land-cover summary, not evidence of realized habitat use by deer.

Although the 10 m annual product provides consistent public coverage through 2025, it can still be affected by seasonal vegetation, crop phenology, cloud or shadow contamination, and spectral similarity among open grassland, shrubland and cropland. The mapped classes should therefore be treated as remote-sensing labels rather than field-verified fine habitat types [13,15].

The temporary increase in edge density and decline in largest-patch index in 2021 suggested a short-term change in mapped landscape structure rather than a persistent loss of vegetation-related cover. This interpretation remains cautious because patch metrics are sensitive to classification resolution, connectivity rules and the grouping of land-cover classes [28].

Taken together, the records did not follow a single temporal trajectory: vegetation-related land cover changed modestly, monitoring counts fluctuated, and releases and detected mortality continued during the study period. Mapped cover, animal counts and management events therefore provide complementary but non-equivalent information for recovery assessment. These parallel records do not establish that land-cover change caused the observed count fluctuations.

### 4.2. Monitoring Records and Management Implications

These records place fluctuations in monitoring counts within an actively managed reserve context. Releases should be treated as management inputs rather than direct evidence of release success, because standardized records of individual survival, reproduction and post-release movements were unavailable [6,7,8,9]. Previous work in Datian also identified Burmese python predation as a conservation concern [30]. In the present study, incomplete event coordinates prevented a spatial analysis of recorded deaths.

Releases can raise the number of individuals in the short term, but their long-term contribution depends on post-release survival, reproduction and integration into the resident population [6,7,8,9]. Mortality records are also affected by detection and reporting, so the high share of python predation should be interpreted as a recurrent concern among recorded deaths rather than as the proportion of all deaths caused by pythons. The available records cannot separate the contributions of releases, predation, survey conditions and land-cover change to fluctuations in counts.

Given these data limitations, management should move from reporting population numbers alone toward integrated monitoring of land-cover dynamics, supplementation, recorded causes of death and survey standardization. Annual composites from a consistent public land-cover product can be used to track trees, grass, shrub and scrub, crops and built area inside the redline and fence, with field plots or drone imagery used to verify key patches [15,16]. The 0–5 km external rings can be retained as an external land-use observation zone, particularly for crop and built-area expansion, but should not be interpreted as a measured edge-effect gradient [17,18,29].

In practice, an integrated annual database would be useful. Land-cover maps, line-transect records, camera-trap detections and patrol events could be stored in a common spatial framework. Each release should include information on individual origin, sex, age, health condition, release site and subsequent observations; each mortality or abnormal event should include date, location, evidence and probable cause. After several years, such records would support a transition from descriptive assessment to spatial evaluation of release performance and detected mortality patterns [6,7,8,9].

### 4.3. Limitations

**Figure 9:**
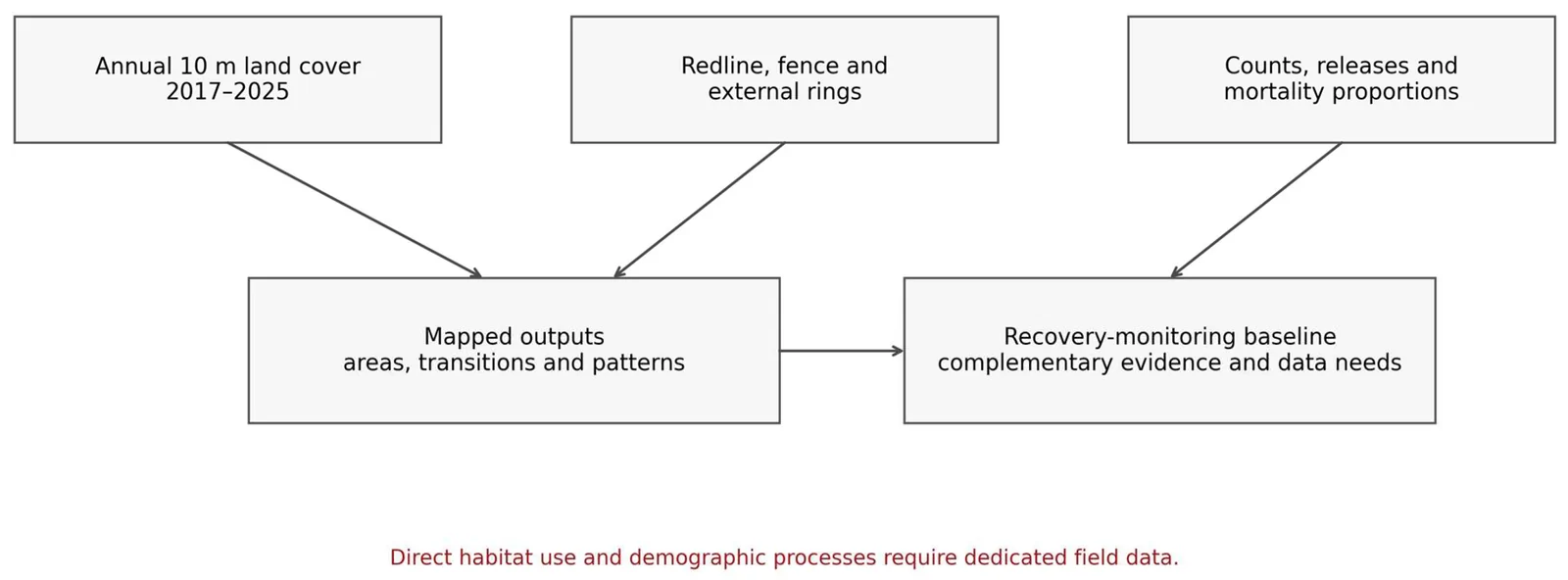
Integrated assessment framework for recovery monitoring. Land-cover, spatial and management records provide complementary evidence; direct habitat use and demographic processes require dedicated field data.

Several limitations remain. The absence of a systematic occurrence dataset precludes analysis of realized habitat selection; incomplete release and mortality coordinates prevent evaluation of release-site suitability and mortality hotspots; and inconsistent survey days and methods limit population-dynamic inference. The 10 m annual product provides public coverage through 2025, but no reserve-specific classification-accuracy assessment was available. Contemporary fence specifications, survey coverage and spatially explicit management-treatment records were also unavailable. Historical descriptions and qualitative discussion cannot resolve these gaps. Because absolute mortality counts are not disclosed, readers cannot independently assess the sampling precision of the reported cause proportion. The assessment therefore provides a reserve-scale baseline for recovery monitoring while leaving direct habitat-use and demographic questions to future field studies. The integrated assessment framework is summarized in Figure 9.

## 5. Conclusions

The combined area mapped as trees, grass and shrub/scrub inside Datian changed little between 2017 and 2025, although its composition varied and mapped grass cover declined. The surrounding area retained much larger proportions of crops and built land. Monitoring counts fluctuated during the same period, alongside post-survey releases and recurrent probable python predation among recorded deaths. Mapped land cover, animal counts and management events are complementary but non-equivalent components of recovery assessment. Their integration provides a practical baseline for recovery monitoring in Datian and other small reserves with uneven historical records. Standardized occurrence and demographic data are still required to assess habitat use and the processes underlying population change.

## Figures and Tables

**Table 1:** Data sources, temporal coverage and analytical use in this study.

Data Type	Source	Period	Use
Land use/land cover	Dynamic World V1 10 m annual composites	2017–2025	Annual land-cover maps, area statistics, transition matrices and exploratory screening indices
Reserve redline	Vector boundary of Datian National Nature Reserve	Fixed boundary	Core spatial unit and surrounding land-use rings
Management fence	Vector boundary of the Datian fence	Fixed boundary	Land-cover composition inside the supplied management boundary
Population monitoring	Reserve management records	2017, 2018, 2019, 2021, 2024 and 2025	Description of annual monitoring-count fluctuations only
Release and mortality records	Reserve management records	Releases: 2019 and 2024; mortality: 2020–2025	Description of supplementation and recorded causes of death

**Table 2:** Rule-based scores assigned to land-cover classes for the exploratory screening index.

Dynamic World Class	Main Score	Conservative Score	Reason for Relative Ordering
Water	0.50	0.40	Potential resource, but not terrestrial cover
Trees	0.70	0.60	Cover value possible; use not established from the map
Grass	0.85	0.75	Open herbaceous cover relevant to foraging
Flooded vegetation	0.65	0.55	Vegetated but hydrologically constrained
Crops	0.25	0.20	Human-managed land-cover class
Shrub and scrub	0.80	0.70	Open and shrub mosaic relevant to management
Built area	0.05	0.05	Intensive human land use
Bare ground	0.10	0.05	Little mapped vegetation
Snow and ice	0.00	0.00	Absent and ecologically irrelevant in this setting

**Table 3:** Focal-year land-cover areas inside the reserve redline (ha).

Year	Water	Trees	Grass	Shrub/Scrub	Crops	Built	Other ᵃ	Total
2017	17.61	1014.33	155.70	10.90	93.19	18.07	0.08	1309.87
2021	16.18	952.61	114.43	85.41	120.59	20.58	0.08	1309.87
2025	15.20	1014.47	92.86	62.97	104.60	19.76	0.02	1309.87

^a^ Other comprises flooded vegetation, bare ground, and snow and ice; snow and ice were absent.

**Table 4:** Staged changes in vegetation-related cover, crops, built area and the exploratory screening index across spatial units.

Spatial Unit	Period	Vegetation Start (ha)	Vegetation End (ha)	Vegetation Change (ha)	Crop Change (ha)	Built Change (ha)	Main Index	Index Change
Inside redline	2017–2021	1180.92	1152.45	−28.47	+27.40	+2.50	0.675→0.666	−0.009
Inside redline	2021–2025	1152.45	1170.29	+17.84	−15.99	−0.81	0.666→0.667	+0.002
Inside redline	2017–2025	1180.92	1170.29	−10.63	+11.41	+1.69	0.675→0.667	−0.008
Inside fence	2017–2021	1174.07	1147.94	−26.14	+26.83	+1.50	0.706→0.697	−0.009
Inside fence	2021–2025	1147.94	1164.90	+16.96	−15.56	−0.31	0.697→0.699	+0.002
Inside fence	2017–2025	1174.07	1164.90	−9.17	+11.27	+1.19	0.706→0.699	−0.007
0–1 km ring	2017–2021	611.87	462.49	−149.39	+132.77	+20.99	0.389→0.348	−0.042
0–1 km ring	2021–2025	462.49	373.69	−88.79	+63.32	+15.91	0.348→0.326	−0.021
0–1 km ring	2017–2025	611.87	373.69	−238.18	+196.09	+36.90	0.389→0.326	−0.063
1–3 km ring	2017–2021	2060.30	1332.06	−728.24	+614.84	+140.07	0.421→0.353	−0.069
1–3 km ring	2021–2025	1332.06	1002.32	−329.74	+382.24	−56.47	0.353→0.327	−0.026
1–3 km ring	2017–2025	2060.30	1002.32	−1057.99	+997.08	+83.59	0.421→0.327	−0.094
3–5 km ring	2017–2021	3520.36	2965.95	−554.41	+638.41	+122.56	0.472→0.427	−0.045
3–5 km ring	2021–2025	2965.95	2503.58	−462.38	+400.79	−43.61	0.427→0.404	−0.022
3–5 km ring	2017–2025	3520.36	2503.58	−1016.78	+1039.20	+78.95	0.472→0.404	−0.067

Vegetation-related cover combines trees, grass and shrub/scrub. Changes were calculated from unrounded areas and indices; displayed endpoints may therefore differ slightly from displayed changes.

**Table 5:** Dominant land-cover transition pathways and areas inside the reserve redline (ha).

Period	Trees→Crops	Crops→Trees	Grass→Shrub	Shrub→Trees	Stable Trees	Stable Crops
2017–2021	21.01	8.32	42.95	2.53	933.70	71.85
2021–2025	10.73	18.97	19.95	41.74	926.67	81.38
2017–2025	14.88	14.66	28.17	3.58	948.08	70.64

**Table 6:** Sensitivity check of the exploratory land-cover screening index inside the reserve redline.

Scoring Scheme	2017	2021	2025	2017–2021 Change	2021–2025 Change
Main scheme	0.675	0.666	0.667	−0.009	+0.002
Conservative scheme	0.580	0.572	0.573	−0.008	+0.001
Vegetation-related proportion	0.902	0.880	0.893	−0.022	+0.014

Changes were calculated from unrounded values; displayed endpoints may therefore differ slightly from displayed changes.

**Table 7:** Landscape metrics of vegetation-related cover inside the reserve redline.

Year	Patch Number	Vegetation-Related Cover (ha)	Mean Patch Area (ha)	Largest Patch (ha)	Largest Patch Index (%)	Edge Density (m/ha)
2017	35	1180.92	33.74	1179.10	90.02	38.36
2021	33	1152.45	34.92	1150.31	87.82	42.45
2025	20	1170.29	58.51	1167.73	89.15	40.37

**Table 8:** Population monitoring and post-survey releases during 2017–2025.

Year	Monitoring Count	Survey Days	Reported Approach	Release After Survey
2017	363	11	SC; LT	0
2018	425	33	SC; LT	0
2019	345	36	SC; LT	46
2020	–	–	–	0
2021	336	23	SC; LT	0
2022	–	–	–	0
2023	–	–	–	0
2024	352	37	SC; LT; IR; UAV	47
2025	371	19	SC; LT; IR; UAV	0

Survey periods fell between March and May in the six years with counts. Survey days are reported duration, not observer-days. SC, synchronous counts; LT, line-transect work; IR, infrared cameras; UAV, drone thermal imaging. A dash indicates unavailable data, and releases occurred after the same-year survey. Across 2020–2025, probable python predation accounted for 68.3% of recorded deaths. This pooled proportion describes detected deaths and is not a cause-specific mortality rate; exact mortality counts are not reproduced.

## Data Availability

Dynamic World land-cover data are publicly available. The export template and aggregate land-cover tables are supplied in Supplementary Materials. The reserve boundary vectors and management records are not publicly available because of species-sensitivity and reserve-management requirements. Exact mortality counts are not reproduced because the reserve administration advised caution regarding their disclosure; only the pooled cause proportion is reported. Access to restricted records may be requested from the corresponding author and remains subject to permission from the Hainan Datian National Nature Reserve Administration.

## Supplementary Materials

The following supporting information can be downloaded at: https://www.mdpi.com/article/10.3390/ani16193086/s1.
